# Oxidative Stress and Dietary Fat Type in Relation to Periodontal Disease

**DOI:** 10.3390/antiox4020322

**Published:** 2015-04-28

**Authors:** Alfonso Varela-López, José L. Quiles, Mario Cordero, Francesca Giampieri, Pedro Bullón

**Affiliations:** 1 Institute of Nutrition and Food Technology “José Mataix Verdú”, Biomedical Research Center (CIBM), University of Granada, Avda. del Conocimiento s.n., Armilla, Granada 18100, Spain; E-Mails: alvarela@ugr.es (A.V.-L.); jlquiles@ugr.es (J.L.Q.); 2 Department of Periodontology, Dental School, University of Sevilla, C/Avicena s.n., Sevilla 41009, Spain; E-Mail: mdcormor@us.es; 3 Department of Clinical Sciences, Marche Polytechnic University, Ancona 60100, Italy; E-Mail: f.giampieri@univpm.it

**Keywords:** periodontitis, lipids, fatty acids, cholesterol, diet, dietary fats, nutrition

## Abstract

Oxidative stress is one of the main factors studied to explain the pathophysiological mechanisms of inflammatory conditions, such as periodontitis. In this respect, nutrition may be of great importance. Actually, research on nutrients’ effects on periodontal diseases has expanded to include those influencing the redox status, which correlates to the inflammatory process. Dietary fat or lipids are often blamed as the major source of excess energy. Consequently, when caloric intake exceeds energy expenditure, the resultant substrate-induced increase in citric acid cycle activity generates an excess of reactive oxygen species (ROS). In addition, dietary fatty acid intake influences in relative fatty acid composition of biological membranes determining its susceptibility to oxidative alterations. From this standpoint, here, we reviewed studies analyzing the dietary fat role in periodontal disease. Research data suggest that periodontal health could be achieved by main dietary strategies which include substitution of saturated fats with monounsaturated fatty acids (MUFA) and polyunsaturated fatty acids (PUFA), particularly *n*-3 PUFA. Maybe in the future, we should analyze the diet and provide some advice to periodontitis patients to improve treatment outcomes.

## 1. Introduction

One of the main open questions regarding periodontal diseases is to know all the aetiological and pathogenic processes involved. The tendency in medical including dental research has been and largely remains to be concentrated on an individual factor, for example, microbial or host. Accordingly, 20th century researchers tried to explain common periodontal diseases with the plaque theory. Based on it, all periodontal treatments are focused on its control and the restoration of the damage it is presumed to have caused. Nowadays, we have obtained good outcomes in periodontal disease control, but it is critical to recognize important limitations: a success rate of 100% is hardly attainable, it is difficult to prevent the establishment of the disease, the identification of the susceptible population is not feasible, and a complete regeneration of the affected tissues, similarly, is presently impracticable. Also, in some forms of periodontal disease, such as aggressive periodontitis, plaque is not essential, and in some patients the disease has a different course depending on the systemic health. Therefore, in the last few years, the importance of the host response has increased markedly [1,2,3]. In this respect, nutrition may be of great importance since it has been implicated in a number of inflammatory diseases and conditions, including cardiovascular diseases, type 2 diabetes mellitus, rheumatoid arthritis and inflammatory bowel disease, all of which have been associated with periodontitis [4].

Nutrition has important long-term consequences for health that contributes to the development and progression of chronic diseases. Traditionally, much of the research on diet and periodontal diseases is focused on only a few nutrients with well-established roles in the formation and maintenance of structural components of oral tissue such as collagen (Vitamin C) and bone (calcium), integrity of epithelial tissue (Vitamin A), or in promoting the formation of plaque that harbours periodontal pathogens (carbohydrates) [5]. More recently, research on nutrients effects on periodontal diseases has expanded to include those influencing redox status, which correlates to inflammatory process. Among other nutrients, the implications of dietary fat on periodontal disease has been analyzed from this perspective. Consequently, in this review, we collect research on periodontal disease and fat, both in humans and animals, with the aim to clarify the possible influence of these components of the diet and establish the most appropriate advice for prevention and treatment of this disease.

## 2. Periodontal Disease and Oxidative Stress

Oxidative stress, defined as a persistent imbalance between the production of highly reactive molecular species and anti-oxidant defences [6], is one of the main factors studied to explain the pathophysiological mechanisms of inflammatory conditions, such as periodontitis [7]. Although the role of oxidative stress in periodontal breakdown is complex, there is increasing evidence for compromised anti-oxidant capacity in periodontal tissues and fluids independent of smoking, and increased advanced glycation end-products levels both in persons with type 2 diabetes and in smokers, which are known risk factors for periodontitis [8]. Such oxidation products can increase neutrophil adhesion, chemotaxis, and priming in hyper-reactive neutrophils, and might augment the damaging effects of the resultant oxidative stress [9,10,11]. In addition to this, the up-regulation of pro-inflammatory transcription factors, such as nuclear factor κB (NF-κB) and activating protein-1 (AP-1), in inflamed periodontal tissues contributes to reduced glutathione depletion and reactive oxygen species (ROS) generation [12,13]. It has been proposed that peripheral blood neutrophil hyperactivity in chronic and aggressive periodontitis exists as a constitutional element [14,15], rather than being entirely the result of peripheral priming by cytokines or plaque bacterial lipopolysaccharide (LPS) [7]. In addition, there may be possible baseline hyperactivity, with low-level extracellular ROS release in the absence of any exogenous stimulus in persons with periodontitis [15,16,17,18].

On the other hand, periodontitis also can influence on serum and/or plasma oxidative markers in humans. Several studies have demonstrated an increase in products of oxidative damage in peripheral blood from persons with periodontitis compared with control individuals [19,20,21]. Furthermore, different evaluations have shown a decreased anti-oxidant capacity in persons with periodontitis [9,11,22,23,24,25,26,27,28]. Actually, there is consistent and strong epidemiologic evidence that periodontitis imparts increased risk for future cardiovascular disease [29] and a cause/effect relationship has been searched; the key question is what the reason for this association is. The presence of a high expression of the receptor for advanced glycation end-products (RAGE) in periodontal tissues [30] is an important finding supporting the sensitivity of these tissues to products derived from oxidative damage. Moreover, advanced glycation end-products, important markers for oxidative stress [31], may promote apoptosis in osteoblasts [32] and fibroblasts [33], and this might have an influence on alveolar bone homeostasis and the progression of periodontitis.

## 3. Dietary Fat and Oxidative Stress

Lipids or fats consist of numerous water-insoluble compounds, including monoglycerides, digycerides, triglycerides, phosphatides, cerebrosides, sterols, terpenes, fatty alcohols, and fatty acids (FAs) [34]. These chemical compounds represent more than 50% of plasmatic membrane and their quantity and quality are known to have an impact on the composition, characteristics and functions of the biological membrane [35,36]. The predominant membrane lipids are phospholipids, which consist in esters of FAs. Additionally, FAs also constitute the main component of phospholipids, triglycerides, diglycerides, monoglycerides, and sterol esters [34]. In particular, triglycerides (esters of FAs with glycerol) represent the foundation of oils and fats (up to 99%) [37]. FAs can vary depending on the chain length, normally between 2 and 22 carbon atoms. It could be a short- (2 to 6 carbon atoms), medium- (8 to 10) or long-chain (12 or more) FA. They also vary depending on the degree of unsaturation of the FA, and may be: saturated FAs (SFA) whose bonds of the free carbon atoms are fully saturated with hydrogen atoms; monounsaturated FAs (MUFA), with a double bond, that is, two adjacent carbon atoms that are not saturated with hydrogen; or polyunsaturated FAs (PUFA), with two or more double bonds. Unsaturated long-chain FAs can be classified into different series according to the position of the last double bond from their terminal methylcarbon that is designated as *n* or ω. Each of these series begins with a precursor: in the case of *n*-3 is α-linolenic acid (ALA); for *n*-6 is linoleic acid, and for *n*-9, oleic acid. From these precursors, by successive elongation and increases in double bonds (by elongase and desaturase enzymes) longer and more unsaturated FAs are formed. The serial number indicates that all FAs within a range have a double bond in the corresponding carbon, counting from the first one on the left and including the group called methyl. Phospholipid’s acyl chain normally varies from 14 to 22 carbons in length and they are either saturated, monounsaturated or polyunsaturated hydrocarbons chains [38]. Long-chain FAs included in *n*-3 and *n*-6 series also constitute eicosanoids (prostaglandins, prostacyclins, thromboxanes, leukotrienes, *etc.*) that are virtually involved in all tissues and organ systems where they perform multiple functions [39].

From the nutritional standpoint, the SFA are mainly found in fats of animal origin, some ground vegetable fats (coconut and palm) and in margarine. MUFA, mostly represented by the oleic acid, are present in large quantities in olive oil, rapeseed oil and in some genetically engineered seeds. Among the polyunsaturated linoleic acid, omega-6 series, is the majority among seed oils (sunflower, corn, soybean, grape, *etc.*). Meanwhile, the polyunsaturated ω-3 series are usually present in the fat of fish and the majority is eicosapentaenoic acid (EPA), which has 20 carbon atoms and five double bonds, and docosahexaenoic acid (DHA), with 22 carbon atoms and six double bonds [40]. Of these FAs, there are two that are called essential because they cannot be synthesized by the body, being necessary to provide them through the diet. These are linoleic acid (*n*-6) and α-linolenic (*n*-3). The first comes from seeds and the second is present, for example, in soybeans. From α-linolenic, the aforementioned EPA and DHA are formed [41].

As mentioned, membrane lipids influence several biochemical parameters, especially at the mitochondrial membrane level [38,42,43]. FAs as components of biological membranes strongly influence membrane fluidity, which, in turn, may influence many physiological processes involved in cell death and survival such as signal transduction, protein import, membrane receptor function and metabolite transport [44]. In addition, the chemical reactivity of the FAs from phospholipids is the main factor responsible for the membrane’s susceptibility to oxidative alterations [38]. This is particularly important because of oxygen, and free radicals are more soluble in the fluid lipid bilayer than in the aqueous solution, focusing on these organic regions and becoming membrane lipids’ primary target of oxidative damage [45]. The sensitivity of biological membranes to oxidative stress is due to the presence of a double carbon-carbon bond in the lipid tails of its phospholipids due to the presence of highly unstable electrons near these bonds [20,46,47,48]. For this reason, polyunsaturated fatty acyl chains of phospholipids from biological membranes are very sensitive to oxidation by ROS, increasing exponentially as a function of the number of double bonds per FA molecule. In turn, ROS lead to carbon-centred radicals within the membranes, resulting in peroxidation of FAs [49]. Therefore, PUFA side chains are much more easily attacked by radicals than saturated or monounsaturated side chains [50]. Oxidative damage to membrane lipids may be directly generated by ROS as hydroxyl radicals or the superoxide anion, or indirectly by some products of lipid peroxidation such as some highly reactive aldehydes [51]. Finally, oxidative damage of membrane lipids leads to its alteration and to changes in membrane fluidity and, in conclusion, alterations in membrane function [49]. Moreover, lipid peroxidation products can produce protein covalent modifications [52].

The importance of dietary FAs resides in the fact that biological membranes, including mitochondrial membrane, adapt their lipid composition to some extent in response to dietary lipid [53,54,55]. In other words, dietary FA intake influences in relative FA composition of the membrane. Thus, humans and animals fed on diets based on olive oil have membranes richer in oleic acid than those fed on diets based on sunflower oil, whose membranes are richer in linoleic acid. Further, adaptations of the electron transport system in response to the type of dietary fat have been widely reported [56,57,58]. In this sense, a polyunsaturated fat source will lead to membranes becoming more prone to oxidation than a saturated or a monounsaturated source, since a low level of FA unsaturation in cellular membranes will decrease cellular oxidative stress. Studies support this idea since their results indicate that experimentally induced decreases in liver and brain FA unsaturation also lower oxidative damage in mitochondrial DNA (mtDNA) [59]. The high concentration of unsaturated FAs in cellular membrane phospholipids not only makes them more sensitive to oxidation reactions, but also enables them to participate in long free radical chain reactions. Thus, a low degree of FA unsaturation in biological membranes may decrease their sensitivity to lipid peroxidation, which can even protect other molecules against lipoxidation-derived damage [42]. This has been widely demonstrated under a wide range of physiological and pathological situations using both animal models and humans [60,61,62,63,64]. Regarding SFA, these acids induce a decrease of membrane fluidity and permeability. An excess of SFA affects cell membrane activity and in particular plasma membrane receptors, which play an essential role in glucose and lipid metabolism [65]. The complexity of SFA actions and the possible differences among different individual SFA suggest that more research is necessary to better understand the role of SFA and their effects in dietary products [66].

In spite of these findings, the role of elongase and desaturase system through the inhibition of the various components of the different series of FAs must be also considered in relation to FA composition of biological membranes [43,67,68]. As is well known, a fine control in the elongation and desaturation pathways is crucial for maintaining the membrane function through its effect on fluidity, or certain signaling cascades’ mediators [69]. Several studies showed that diet rich in MUFAs (from olive oil) stimulates the formation of membranes where the presence of *n*-3 PUFAs when compared with diets rich in *n*-6 PUFAs (based on sunflower oil as fat source) are increased [70,71]. Likewise, a shift in the proportion of *n*-6 PUFAs with *n*-3 PUFA-rich diets was observed too. There is a ratio between *n*-9/*n*-6 series suggesting that FA from *n*-9 series could be more easily incorporated in detriment to *n*-6 series. Diets rich in unsaturated FAs increase levels of MUFAs and rich in *n*-3 PUFAs increase *n*-3/*n*-6 ratio [72,73]. The maintenance of membrane fluidity is achieved in mammals by a homeostatic mechanism and compensation control that maintains an optimal relationship between the proportion of saturated and unsaturated FAs [74,75]. This mechanism would explain that no significant changes were observed in saturation of membrane lipids since an increase in the *n*-6 series resulted in a decrease in the *n*-3 and *vice versa* [43,53]. Animals with a high longevity have a low degree of membrane FA desaturation based in the redistribution between types of PUFAs without alteration in the total PUFAs’ content and phospholipids redistribution [50]. There is a tendency that the proportion of SFAs remains stable, as C18:0, which is largely dependent on the concentration needs of each tissue. However, in the case of C16:0, adaptability is observed according to the composition of the diet, with a special ability to activate the Δ9 desaturase [38]. In diets rich in FAs of the *n*-9 series, an increase in MUFAs is shown, mainly when oleic acid is administered [64]. In the case of the *n*-6 series, their membrane levels only seem to increase when a large number of their components is administered in the diet [76]. The *n*-3 series is present when good amounts is administered, or when there is a deficiency of *n*-6, after the two series have competed the Δ6 desaturase, which determines the ratio *n*-3/*n*-6 [77]. It was suggested, however, that high intakes of linoleic acid, even at current levels, were harmful and should be reduced [78]. This theoretical argument is based on the possibility that *n*-6 and *n*-3 PUFA in the diet could compete with each other in elongation and desaturation pathways and that longer-chain *n*-6 PUFA are precursors to pro-inflammatory eicosanoids, which lastly enhance oxidative molecular species production. Also, high susceptibility to oxidation of this edible oil has prompted many warnings concerning benefits to health [79]. In addition, it is important to point that dietary lipids are often blamed as the major source of excess energy, although it is difficult to differentiate the effects of dietary fat and other energy nutrients independent of total energy intake [80]. When caloric intake exceeds energy expenditure, the resultant substrate-induced increase in citric acid cycle activity generates an excess of ROS [81]. In turn, oxidative stress alters intracellular signalling pathways [76] leading to more oxidative stress generation among other harmful consequences. At least that has been noted in some tissues as in a murine model where a high fat-diet has been associated with up-regulation of pathways for ROS production and oxidative stress in both the liver and adipose tissue, which was followed by an elevation of tumor necrosis factor-α (TNF-α) and free FAs in the plasma and liver [82].

## 4. Dietary Lipids and Periodontitis

There are many reports focused on the relationship of lipids with periodontal disease both, in animals and humans, which are presented in Table 1 and Table 2, respectively. Research on this relationship can be clearly differentiated since it analyzes the role of dietary lipids on periodontal disease from a quantitative or qualitative standpoint. Specifically, studies usually evaluate the possible effect of diets with different lipid content or FA profile in the diet. At the moment, it is difficult to find human studies that analyze the total dietary amount of this macronutrient on periodontitis by total intake estimates or experimental interventions. In turn, there is much information from dietary interventions in animals, particularly rats [83,84,85,86] and mice [87], although rabbits have been used too [88]. Almost all studies have increased the dietary fat amount, usually by increasing saturated fat-rich foods [86,87], adding cholesterol [85,86] or both [88], to induce atherosclerosis [88], obesity [89] or/and diabetes, in many cases due to energy intake increase. Feeding rats with a high-cholesterol diet induced an increase in blood total cholesterol and a decrease in high-density lipoprotein (HDL)-cholesterol [86]. These chronic diseases have been largely associated with periodontal diseases and there are many reviews on this topic [7,90,91,92,93,94]. Several studies have reported associations between consumption of those experimental diets and outcomes related to higher alveolar bone destruction [84,85] or inflammation [88]. Others have combined dietary treatments with additional methodologies to evaluate the effect of the diet on the biological response to periodontal microorganisms. In rats, high cholesterol diets enhanced proliferation of the junctional epithelium with increasing bone resorption and cell-proliferative activity associated with application of bacterial LPSs and proteases to the gingival sulcus [89]. Excessive intake of this type of nutrients has negative effects on periodontal health, especially if they include saturated fat or cholesterol. Several markers of inflammation or oxidative stress further are elevated when measured at a local or systemic level including 8-hydroxy-2′-deoxyguanosine (8-OHdG) and depletion of reduced glutathione content (represented by low reduced glutathione/oxidized glutathione ratios) [83,85,95]. Similarly, the effects of bacterial pathogens and their products on production of pro-inflammatory cytokines in fibroblasts were augmented [95].

**Table 1:** Human studies on lipids effects on periodontitis.

Study Type (Duration)	Subjects	Age (*n*)	Main Clinical Outcomes/Periodontitis Definition	Main Results/Conclusions	Reference
Cross-sectional	NHANES 1999–2004 participants (USA)	≥20 years (9182)	Periodontitis: PPD ≥ 4 mm & AL ≥ 3 mm in any mid-facial or mesial tooth	Inverse association of *n*-3 PUFA, DHA, EPA & GLA intake with periodontitis prevalence	[96]
Cross-sectional	Patients attended the Sevilla University Dental School (Spain)	≥35 years (56)	Periodontitis: AL ≥ 6 mm in ≥ 2 teeth & ≥ 1 sites with PPD 5 ≥ mm	Serum levels of *n*-6 PUFA, SFA and MUFA were higher in the periodontitis group compared to subjects without periodontitis which also occurred with peroxida bility index	[97]
Cross-sectional	Patient form dental school of the Rio de Janeiro State University (Brazil).	46.0 ± 8.8/31.5 ± 7.5 years (37)	chronic generalized periodontitis & gingivitis were diagnosed according to criteria described by the American Academy of Periodontology	Higher serum levels of DHA, DPA, EPA, & AA were observed in patients with chronic generalized periodontitis when compared with patients with gingivitis	[98]
Cohort (5 years)	Niigata study participants (Japan).	74 years (55)	Periodontal disease events: *n*° of teeth with AL ≥ 3mm/year	Negative association of DHA intake with risk of periodontal disease events	[99]
Cohort (3 years)	Niigata study participants (Japan)	75 years (235)	Periodontal disease events: *n*° of teeth with AL ≥ 3mm/ year	Positive association of *n*-6/*n*-3 PUFA ratio with risk of periodontal disease events	[100]
Cohort (3 years)	Niigata study participants (Japan)	75 years (264)	Periodontal disease events: *n*° of teeth with AL ≥ 3mm/year	Positive association of SFA intakes ratio with risk of periodontal disease events in non-smokers	[101]
Randomized controlled trial (DB) (12 weeks)	Subjects with periodontitis (USA)	18–60 years (30)	MGI, PI, PPD	Supplementation with borage oil (a GLA source) or EPA improved PPD, but only the first was statistical significant respect to the placebo (an olive & corn oil mixture). Additionally, it was the only that also improved MGI.	[102]
Randomized controlled trial (DB) (3/6 months)	Subjects with advanced untreated chronic periodontitis (Egypt)	30–70 years (80)	PI, MGI, BOP, PPD & CAL	Dietary supplementation with a combination of fish oil (EPA & DHA-rich) & aspirin after SRP, reduced PPD & salivary levels of RANKL & MMP-8 & increased CAL	[103]

Abbreviations: AA: Arachidonic acid; AL: Attachment loss; BOP: Bleeding on probing; CAL: Clinical attachment level; CPI: Community periodontal index; DHA: Docosahexanoic acid; DB: Double-blind; DPA: Docosapentanoic acid; EPA: Eicosapentanoic acid; GLA: *gamma*-linolenic acid; MGI: Modified gingival index; MMP-8: Matrix metalloproteinase-8; MUFA: Monounsaturated fatt acids; NHANES: National Health and Nutrition Examination Survey; PI: Plaque index; PPD: Peridodontal probing depth; PUFA: Polyunsaturated fatty acids; RANKL: Receptor activator of nuclear factor kappa-B ligand; SFA: Saturated fatty acids; SRP: Scaling and root planning; USA: United States of America.

**Table 2:** Animal studies on lipids effects on periodontitis.

Animal Model	Gender Age/Weight (*n*)	Dietary Treatments (Duration)	Periodontal Intervention (Duration)	Main Results/Conclusions	Reference
New Zealand rabbits with dietary-induced atherosclerosis	Male 2.5 kg (48)	CoQ_10_, squalene, or hydroxytyrosol supplements after atherosclerosis induction (30 days)	None	Hydroxytyrosol reduced endothelial activation of gums & squalene additionally decreased fibrosis	[7]
Obese (by diet) & non-obese Wistar rats	Male 8 weeks (42)	High-fat diet combined with exercise training or not (4/8 weeks)	None	Rats fed a high-fat diet showed higher serum ROM & gingival 8-OHdG levels, & gingival GSH/GSSG ratio than rats fed a regular diet that were reduced by exercise training	[83]
Obese (by diet) & non-obese C57BL/6J mice	Both 4 weeks (80)	High-fat or standard diet with or without moderate exercise after obesity development (4 weeks)	*P. gingivalis*-soaked or sterile ligatures (last week)	High-fat diet increased *P. gingivalis*-induced ABL which associated to higher serum levels of TNFα, MCP-1, IL-1β & lower of IL-6 & IL-12p70. However moderate daily exercise decreased ABL & restores cytokines normal levels.	[87]
Wistar rats	Male 8 weeks (24)	high-cholesterol or regular diet (12 weeks)	None	High-cholesterol diet decreased alveolar bone density & increased TRAP–positive osteoclasts & the expression of 8-OHdG in the periodontal tissue	[85]
Wistar rats with & without induced periodontitis	Male 8 weeks (32)	high-cholesterol or regular diet (8 weeks)	Application of LPS & proteases or pyrogen free water (last 4 weeks)	High-cholesterol diet increased proliferation of the junctional epithelium with increasing bone resorption & cell-proliferative activity of the junctional epithelium induced by LPS & proteases.	[86]
Wistar rats with & without induced periodontitis	Male 8 weeks (32)	high-cholesterol or regular diet (8 weeks)	Application of LPS & proteases or pyrogen free water (last 4 weeks)	High-cholesterol diet augmented the induced production of pro-inflammatory cytokines by bacterial products & mitochondrial 8-OHdG in periodontal tissues	[95]
Sprague-Dawley rats with induced periodontitis	Female 8–9 weeks (95)	Diets containing 17% fish oil & 3% corn oil or 5% corn oil only (22 weeks)	Infection with *P. gingivalis* (last 12 weeks)	Rat fed on diets containing fish oil had less ABL	[104]
Sprague-Dawley rats with induced periodontitis	Female 8–9 weeks (82)	Diets containing 17% fish oil & 3% corn oil or 5% corn oil only (22 weeks)	Infection with *P. gingivalis* 381 or A7A1-28 (last 12 weeks)	Diet containing fish oil led to decreased IL-1β, TNF-α & enhanced IFN-γ, CAT & SOD gingival mRNA levels	[105]
BALB/c mice with & without induced periodontitis	Female 6–8 weeks (70)	Diet containing 10% tuna oil or sunola oil (57 days)	Orally inoculation with *P. gingivalis,* with a mixture of *P. gingivalis* & *F. nucleatum* or none (last 43 days)	Diet containing 10% tuna oil decreased ABL in inoculated mice	[106]
Wistar rats	Not given 4 weeks (60)	Diet containing 10% refined fish oil or corn oil (6–8 weeks)	Tooth movement by 20 g continuous force on the lingual side of 1^st^ maxillary molars with a lateral expansion spring (0/3/7/14 from the sixth week)	The diet containing 10% fish oil reduced tooth movement, *n*° osteoclasts & bone resorption	[107]
Sprague-Dawley rats with & without induced periodontitis	Male Adults (39)	Orally gavaged with *n*-3 PUFA (EPA + DHA) or saline supplements (15 days)	LPS or saline injections	*n*-3 PUFA supplements increased IL-1β & OC serum levels in LPS-treated rats	[108]
Aged & young Wistar rats	Male 80–90 g (72)	Virgin olive*,* sunflower or fish oil, as life-long dietary fat sources (6/24 months)	None	At endpoint, virgin olive oil fed rats showed the lowest age-related ABL, followed by those fed on fish oil. Additionally, sunflower oil fed rats showed a high degree of fibrosis & a moderate degree of inflammation	[109]
Wistar rats	Male 60 days (28)	Hyperlipidic or standard diet (17 weeks)	None	The hyperlipidic diet consumption led to development of more periodontal disease sites defined by an ABL > 0.51 mm (75th percentile)	[84]

Abbreviations: 8-OHdG: 8-Hydroxydeoxyguanosine; ABL: Alveolar bone loss; BOP: Bleeding on probing; CAT: Catalase; CoQ_10_; Coenzime Q_10_; COX-2: Cicloxigenase-2; DHA: Docosahexanoic acid; EPA: Eicosapentanoic acid; *F. nucleatum: Fusobacterium nucleatum*; GSH/GSSG: reduced glutathione/oxidized glutathione ratio; IFN-γ: Interferon gamma; IL-12p70: Interleukin-12 subunit p70; IL-1β: Interleukin 1-beta; IL-6: Interleukin-6; LPS: Lipopolysaccharide; MCP-1: Monocyte chemotactic protein-1; OC: Osteocalcin; *P. gingivalis: Porphyromonas gingivalis;* PUFA: Polyunsaturated fatty acids; ROM: Reactive oxygen metabolites; SOD: Superoxide dismutase; TNFα: Tumor necrosis factor alpha; TRAP: Tartrate-resistant acid phosphatase.

On the other hand, qualitative aspects of lipids in the diet also have major importance for the severity of periodontal disease. Several studies have investigated the potential effects of different FAs from diet or as supplements, on periodontal disease, both in humans and in animal models. The most important evidence supporting SFA negative effects derived from a longitudinal study initiated in 1998 in Niigata City (Japan), to evaluate the relationship between systemic health and dental diseases [110]. It assessed persons aged 70 years old non hospitalized, nor institutionalized; after five years, a dietary assessment (by self-administered diet history questionnaire) was conducted and dental health of the cohort was followed-up for one year. Eventually, a negative association was found between SFA intake and the number of periodontal disease events [100], although only in non-smokers [101]. These results support a causal association for saturated fat intake on periodontitis progression, but just in older people, so more cohort studies are required to extend that effect to other age groups.

The effects of PUFA on periodontitis, in turn, have received more attention, and to establish conclusions requires making distinctions between *n*-3 and *n*-6 PUFA. Particularly, most studies have focused on the possible positive effects on periodontitis for polyunsaturated FAs belonging to the *n*-3 series, either as preventive or adjunctive during treatment. These FAs include ALA and its derivatives, mainly EPA, and DHA. In a cross-sectional study, Naqvi *et al.* [96] reported negative associations among dietary intakes of those three representative *n*-3 PUFA and periodontitis. As for longitudinal studies, results from the Niigata City study are available too. In these, an inverse independent relationship with periodontal disease progression was found just for energy-adjusted dietary intakes of DHA after controlling for several confounding factors, but not of total *n*-3 PUFA or EPA [99,101]. In addition, there are available data from studies evaluating FAs levels in serum [97,98] but in general they do not clarify the role of the different Fa type in periodontal disease. In one of these, it has been reported higher levels of *n*-6 PUFA, SFA and MUFA than in periodontally healthy persons [97]. In other, serum levels were compared between patients with chronic generalized periodontitis and those displaying only gingivitis. In this, patients with chronic generalized periodontitis had higher levels of arachidonic acid as representative of *n*-6 PUFA but also of *n*-3 PUFA (DHA, DPA and EPA) [98]. This could indicate that higher intake of all these PUFA would facilitate progression of gingivitis to generalized periodontitis. However, calculated ratios between these fatty acids, which seem more important, did not differ between groups [98]. Moreover, gingivitis patients now could show periodontitis in the future. Therefore, to establish any causal relationship, some cohort studies are needed.

Some interventions have evaluated the utility of using supplements of *n*-3 PUFA alone or supplementing periodontal therapy, both in animals and humans. A combination of DHA and EPA as supplement to a regular diet was tested in rats with induced-periodontitis by LPS injections; their use was ineffective in preventing alveolar bone loss, either alone or combined with therapy [103,111]. This is consistent with data from the transversal study using a National Health and Nutrition Examination Survey (NHANES) subset performed by Naqvi *et al*. [96], who noted that associations hardly changed after accounting amounts from supplements containing DHA, EPA, or ALA or after excluding people reporting their use, except for ALA. Despite the absence of bone resorption differences, both treatments with *n*-3 PUFA maintained lower gingival levels of different membrane phospholipid mediators in respect to only endotoxin-treated rats [111]. When rats received prophylactic treatment, levels were similar to healthy controls for prostaglandin E2 (PGE2), leukotriene B4 (LTB4), and plasminogen activator factor (PAF), whereas in rats treated only during periodontitis induction, only PGE2 remained in similar levels although LTB4 levels were lower than in disease controls. On the other hand, serum levels of Interleukin(IL)1-β and osteocalcin are higher in rats receiving either *n*-3 PUFA treatment than in only LPS-treated rats. Curiously, PGF2α decreased in respect to both healthy and diseased rats without treatment, and only in rats with a non-prophylactic *n*-3 PUFA treatment [111]. Serum C-reactive protein (CRP) levels, in turn, did not show differences among groups [108].

In humans, *n*-3 PUFA supplementation effect was investigated in the treatment of experimental gingivitis. Healthy volunteers who discontinued oral hygiene and were treated with fish oil showed a significant decrease in gingival index compared to controls [112]. Additionally, in a parallel double blind clinical study, advanced chronic periodontitis patients daily treated with fish oil and aspirin after scaling and root planning demonstrated reduction in periodontal probing depth and attachment gain after three and six months in the *n*-3 group, compared to baseline and to the placebo-treated group. This suggests that supplementation with *n*-3 PUFA and aspirin resulted in a significant shift in the frequency of periodontal probing depths ≤4 mm. Salivary receptor activator of NF-κ-B ligand (RANKL) and Matrix Metalloproteinase-8 (MMP-8) levels showed significant reductions in the *n*-3 group at three and six months, and compared to the control at six months [103].

Regarding *n*-6 PUFA, those derived from linoleic acid showed harmful effects, if they were consumed at a higher proportion than *n*-3 polyunsaturated acids. Likewise, Iwasaki *et al*. [101], using estimated energy-adjusted dietary intakes, found that a high *n*-6 to *n*-3 PUFA ratio, but not total *n*-6 acids intake, was significantly associated with a greater number of periodontal disease events in the elderly. In fact, authors consider dietary *n*-6 to *n*-3 PUFA ratio as the main predictor to estimate influence on periodontal disease events [101]. An intervention study in humans demonstrated a beneficial effect of *n*-3 and *n*-6 PUFA on periodontitis improving clinical outcomes. Adults with periodontitis received supplementation with olive oil as placebo, fish oil as a source of *n*-3 PUFA (mainly EPA) or borage oil as source of *n*-6 PUFA (mainly γ-linolenic acid, GLA). Improvement in gingival inflammation was observed in subjects treated with borage oil, whereas improvement in probing pocket depth was seen in those subjects treated with either fish oil or borage oil [102].

Animal studies in rodents also suggest beneficial effects of a diet with lower *n*-6 to *n*-3 PUFA ratio. Usually, diets with high content of *n*-3 PUFA use fish oil as fat source, whereas *n*-6 acids-rich diets are more diverse although corn and sunola oil are the most common sources. Despite diet similarities, they differ according to experimental periodontitis induction. Female mice inoculated with bacteria exhibited less alveolar bone loss when fed with tuna oil than with the sunola oil treated group [106]. Likewise, alveolar bone resorption was also reduced in female rats with induced periodontitis by bacterial inoculation treated with fish oil more than with a corn oil-containing diet [105]. In rats, whose teeth were subjected to a continuous force, fed diets containing refined fish oil showed less tooth movement, accompanied by less bone resorption than those fed diet containing corn oil [107]. Genetic expression analyses for several biomarkers (interferon-γ, IL-1β and TNF-α) and different enzymes (cicloxigenase-2, catalase and superoxide dismutase) in gums have suggested that this beneficial is consequence of the modulating effect of *n*-3 PUFA on gingival inflammation [104,105]. Additionally, when EPA, DHA and ALA levels were analyzed in oral soft tissues, they were higher in animal fed diet rather than in fish oil [106]. Periodontal tissue membrane lipid profile changes along with those of inflammation mediators levels in both blood and periodontal tissues, combined with improvements of some periodontal outcomes, suggests that dietary modifications of FAs present in biological membranes might protect periodontal tissues of local and systemic hazards.

Recently, interest in the role of MUFA on periodontitis has surfaced. According to research in rats, MUFA might offer similar or even better advantages than *n*-3 PUFA [109]. In the most recent study, rats were fed three different diets, which contained virgin olive oil, sunflower oil, or fish oil as fat sources, to check their life-long effects. At endpoint, sunflower oil fed rats showed the highest age-related alveolar bone loss, followed by those fed on fish oil. Findings concerning bone resorption markers (RANKL and Osteoprotegerin) could explain these differences at least in part; RANKL expression in gingiva was the highest for virgin olive oil fed young animals, which decreased in old animals. Nevertheless, its circulating levels changed similarly for all dietary treatments. For osteoprotegerin, the lowest genetic expression was found for fish oil fed animals at six months and increased in old animals. On the other hand, animals fed on virgin olive oil showed the lowest concentration of plasma levels at six months, but concentration increased with age in both virgin olive oil and fish oil fed rats. Similarly, in old animals, the highest levels of lipid peroxidation in the gingiva was observed in sunflower oil fed rats, but age differences were only found for virgin olive and fish oil fed animal. Histologically, only the sunflower group showed a high degree of fibrosis and a moderate degree of inflammation at 24 months, although both sunflower and fish oil group showed a reduction in cellularity. Additionally, a wide gene expression analysis on gingival tissue was performed for genes implicated in several processes: inflammation (IL-1β, IL-8, IL-6 and TNF-ɑ), apoptosis (Bad, Bax and Bcl-2), mitochondrial biogenesis (Tfam, PGC1 and Sirt1), mitochondrial autophagy, and antioxidant defense, as well as for electron transport chain Complex I constituents (MT-ND1, MT-ND4 and Ndufs1). Results suggest that MUFA or *n*-3 PUFA positive effects might be because FAs allow mitochondrial to maintain turnover through biogenesis or autophagy, and they seem to induce the corresponding antioxidant systems to counteract age-related oxidative stress, without inhibiting the mitochondrial electron transport chain [105]. In spite of these observations in animals, two nutritional interventions in humans [102,112] where olive oil was used as placebo do not support this idea. Both control groups showed worst outcomes at the end than those treated with *n*-3 PUFA [102,112] or even than *n*-6 PUFA-treated groups [102]. Differences with these trials could be to the use of non-virgin olive oil, so certain natural bioactive compounds from the minor fraction of virgin olive oil, particularly phenolic components, were not present. Among others, hydroxytyrosol has demonstrated important antioxidant and anti-inflammatory properties, which could be of interest for the prevention of diseases traditionally related to oxidative stress, such as cardiovascular diseases and cancer [113]. Among other findings, it has shown to protect LDL particles from lipid peroxidation both *in vitro* [114,115] and *in vivo* [116,117], as well as to inhibit prostaglandin E2 synthesis [118]. Another major compound present in olive oil, squalene [119], also has shown important antioxidant properties *in vitro* [120,121] and it has been suggested that it may have a critical role reducing sunlight-derived free radical oxidative damage to the skin [121,122] since it is one of the main lipids in its surface [123]. Results from an experimental model of atherosclerosis and stetatohepatitis in rabbits (by a 50 days’ treatment with a saturated fat-rich diet with cholesterol) manifested some inflammation features also at gingival level. After development of atherosclerosis, subsequent intervention with hydroxytyrosol reduced endothelial activation and, with squalene, additionally decreased fibrosis. The results suggest that the gingival vascular changes observed after the atherosclerotic diet have been reversed by hydroxytyrosol and squalene [88]. Furthermore, it is important to note that interventions use olive oil as supplemental placebo, and it might not modify the largely lipid profile of diet. This would not markedly increase the content of MUFA in biological membranes, which could be another reason for the positive role of olive oil as a fat source.

## 5. Conclusions

Nowadays, to treat periodontal disease, the treatment approach is based on the concept that the biofilm/bacteria are the main cause. The new thrust in periodontal medicine highlights the implications of systemic health in the homeostatic equilibrium between aggression and host response. Therefore, it is mandatory to know in depth details of the systemic health status of periodontal patients. The desirable outcome would be to recognize general health factors that could influence, if modified, periodontal disease control. It is known that the main systemic diseases such as diabetes and cardiovascular disease can be prevented with the control of risk factors. The diet, especially the lipids, produces an impairment of the general conditions. Some data show that lipids could affect the natural development of periodontal disease. In that sense, animal experiments have shown fat-rich diets have an adverse effect on periodontal health [83,85,86,87,95], which often correlates with a higher degree of oxidative stress [83,85,95]. In all of them, increase of fat was due to increase of saturated fat but it would be interesting to know what would happen with other fat types with different susceptibility to lipid peroxidation. In spite of this appreciation, these diets usually are energy-dense diets, and excessive energy intake also has been associated with high oxidative stress levels [81]. Regarding the type of dietary fat, longitudinal studies in humans would indicate that the dietary *n*-3/*n*-6 PUFA ratio is important [96,100]. This is in line with the fact that, at the moment, studies on *n*-3 PUFA with supplements have not been entirely successful in animals (at least regarding bone loss), although in humans some slightly better clinical outcomes have been obtained [103,112], although the same effect was found across these studies [102]. However, when treatments consisted of diets with high *n*-3/*n*-6 ratios, it was found to clearly have positive results [105,106], but for now this has only been tested in animal models. On the other hand, the role of total intakes of PUFA has been discussed more extensively [97,101]. Lastly, while MUFA susceptibility to oxidation is considered low [42,50], studies evaluating MUFA-rich diets are limited and they offer conflicting results. In any case, based on the significant lack of studies in humans, it is difficult to provide dietary advice. Possibly, in the future, we should analyze the diet and give some advice to patients to improve treatment outcomes.
